# Analysis of the Barley Malt Rootlet Proteome

**DOI:** 10.3390/ijms21010179

**Published:** 2019-12-26

**Authors:** Ramamurthy Mahalingam

**Affiliations:** USDA-ARS, Cereal Crops Research Unit, Madison, WI 53726, USA; mali.mahalingam@usda.gov; Tel.: +1-608-890-0300

**Keywords:** barley, gene ontology, rootlet, jasmonic acid, malting, proteome, secondary metabolism, pathway analysis

## Abstract

Barley seeds are one of the main ingredients of the malting industry for brewing beer. The barley rootlets that are separated from the kilned seeds at the end of the malting process and used as animal feed are one of the byproducts of this industry. In this study, the proteome of rootlets derived from two stages of the malting process, germination and kilning, from a popular malting barley variety were analyzed. A label-free shotgun proteomics strategy was used to identify more than 800 proteins from the barley rootlets. A high coverage and high confidence Gene Ontology annotations of the barley genome was used to facilitate the functional annotation of the proteins that were identified in the rootlets. An analysis of these proteins using Kellogg Encyclopedia of Genes and Genomes (KEGG) and Plant Reactome databases indicated the enrichment of pathways associated with phytohormones, protein biosynthesis, secondary metabolism, and antioxidants. Increased levels of jasmonic acid and auxin in the rootlets further supported the *in silico* analysis. As a rich source of proteins and amino acids use of these by-products of the malting industry for animal feed is validated. This study also indicates rootlets as a potential source of naturally occurring phenylpropanoids and antioxidants that can be further exploited in the development of functional foods.

## 1. Introduction

Barley (*Hordeum vulgare* L.) is the fourth-largest cereal cultivated around the world. Barley is used for feed and in the malting industry. In the United States of America (USA), a significant part of harvested barley is used for malt and beer production. The barley malting process involves three consecutive stages—steeping, germination, and kilning, which have been described in detail [1]. Temporal proteome analyses of the seeds from these different stages have been reported earlier [2]. Malt rootlets, called as chits, are germs that appear during the malting process of barley, which are separated before the brewing process and are used as a byproduct for animal feed.

The extraction of useful compounds from plant byproducts is known for eons. Rosemary, tea, and grape extracts are used as natural antioxidants in foods or food supplement. The production of antioxidant extracts from Durum wheat bran [3], peanut hulls [4], evening primrose [5], citrus peels, and seeds [6] are some examples of natural byproducts that have been reported. In fact, antioxidant activity in the free extract from the malt rootlets has also been reported [7].

Detailed analysis of root proteomes of developing roots of wheat [8], maize [9], and rice [10] have been reported. Root proteomic studies in barley generally have focused on characterizing the alterations in the proteome in response to abiotic stresses, such as salinity [11,12,13,14], drought [15,16], or nutrient deficiency [17]. In this study, the rootlet proteome at two different stages of the malting process—during germination and after kilning were analyzed. The proteome of the kilned rootlets and the kilned seeds without the rootlets were also examined. This barley rootlet proteome analysis aided in identifying the biochemical pathways associated with secondary metabolites that are enriched in this malting industry byproduct.

## 2. Results

### 2.1. Overview of the Proteome Analysis Pipeline

**Figure 1:**
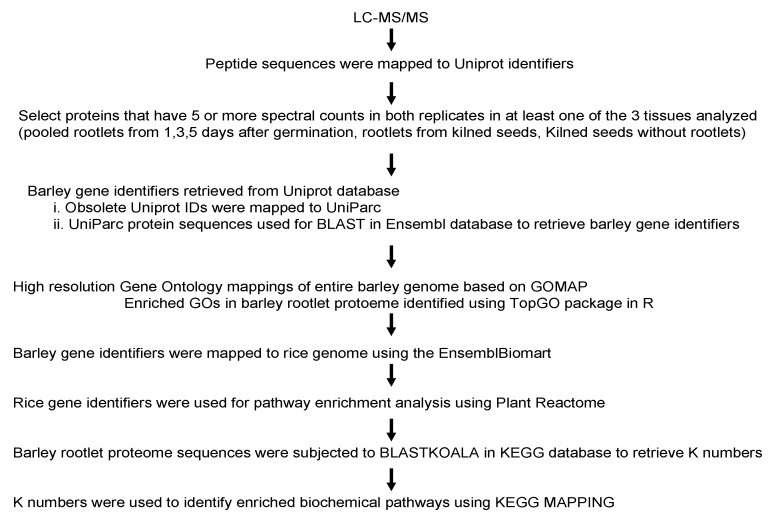
An overview of the various steps in the analysis of the barley rootlet proteome.

The rootlets for this analysis were collected from barley seeds subjected to the malting process. Rootlets were collected one, three, and five days during the germination stage and after the end of the kilning process. An equal amount of proteins from the three different days of germination were pooled together for the proteome analysis. Apart from the rootlets, the proteome of the kilned seeds was also examined. Several strategies were used in order to gain insight into the proteins identified in the rootlets and the kilned seeds (Figure 1). Each of the steps outlined below are described in detail in the subsequent sections. 

### 2.2. Overview of the Mass Spectrometry Data of Barley Rootlets and Kilned Seeds

In the Scaffold software, the entire data set was filtered by setting up a 1% False Discovery Rate (FDR) as threshold for peptide and protein identification, and with at least two peptides. Based on this filtering, 96,100 spectra were analyzed, which led to the identification of 2111 proteins with more than one spectral count, with at least two minimum peptides (Table S1). Following these filtering criteria, the number of spectra that were identified in the two biological replicates from these three tissue samples was comparable. The kiln rootlet samples showed a very tight overlap in the number of spectra identified in the two replicates. The total number of peptides identified in replicates of each tissue varied from 12,048 to 15,768 with the standard deviation ranging between 2.5% and 6.8%, indicating the high reproducibility of peptide counts within biological replicates. The spectral signatures that were mapped to the proteins further supported this (Table 1).

### 2.3. Comparative Analysis of the Rootlet and Kilned Seed Proteome

Several exclusion criteria were applied to define a robust dataset for further detailed analysis. 1. Proteins with less than five spectral counts from the average of the two biological replicates were excluded. 2. Proteins that had less than 10% protein coverage based on the identified peptides in each replicate were not included and 3. When the protein identity probability was less than 98% from each replicate, such proteins were excluded from further analysis. Based on these three criteria, 765 proteins were identified from the rootlets pooled from one, three, and five days after germination (Table S2). In the rootlet samples that were derived from the kilned seeds there were 805 proteins (Table S3) and in the kilned seeds without the roots, 587 proteins were identified (Table S4). Thus, the rootlet proteome is quantitively more complex than the seeds, even after the rigorous filtering of the three data sets.

Of the 765 proteins with more than five spectral counts in the germinating root samples, barley gene identifiers of 363 uniport entries were retrieved from biomart and 400 were obtained by BLAST analysis of the corresponding UniParc identifiers in the Barley Ensembl database. Two entries remained unmapped. There were 711 unique proteins after removing the duplicates. Of the 805 Uniprots with more than five spectral counts, 383 were mapped to barley genes while using Biomart. For the remaining 422 obsolete Uniprots, the corresponding Uniparc protein sequences were retrieved from Uniprot database and were BLAST searched in the barley Ensembl database. Of the 805 uniprots, 111 were identified as being redundant. The 694 Uniprot identifiers were eventually mapped to 683 unique proteins.

**Figure 2:**
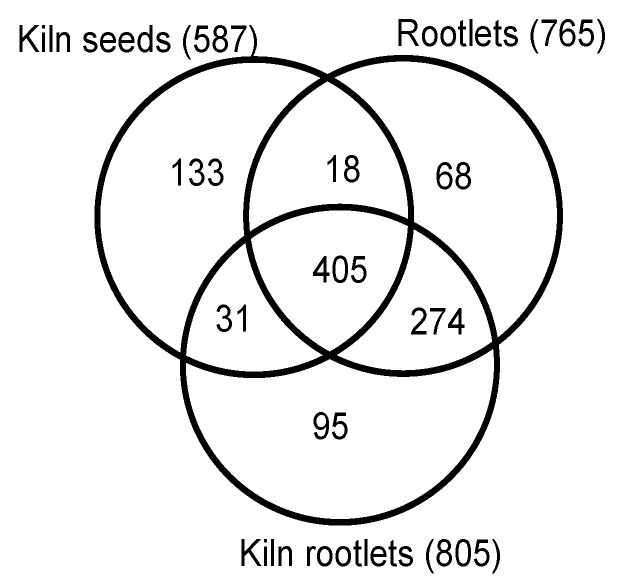
Quantitative comparison of the proteomes of germinating rootlets, kilned seeds, and rootlets of malting barley.

Uniprot protein lists from the germinating rootlets, the kilned rootlets and kilned seeds were compared. A significant overlap of 679 proteins was observed based on the comparison between the germinating rootlets and the kilned rootlets (Figure 2). Interestingly, a similar analysis between the kilned rootlets and the kilned seeds showed that nearly 46% of the kilned rootlet proteome was unique and not represented in the seeds (Figure 2). 

### 2.4. Gene Ontology (GO) Enrichment Analysis of the Rootlet Proteome

**Figure 3:**
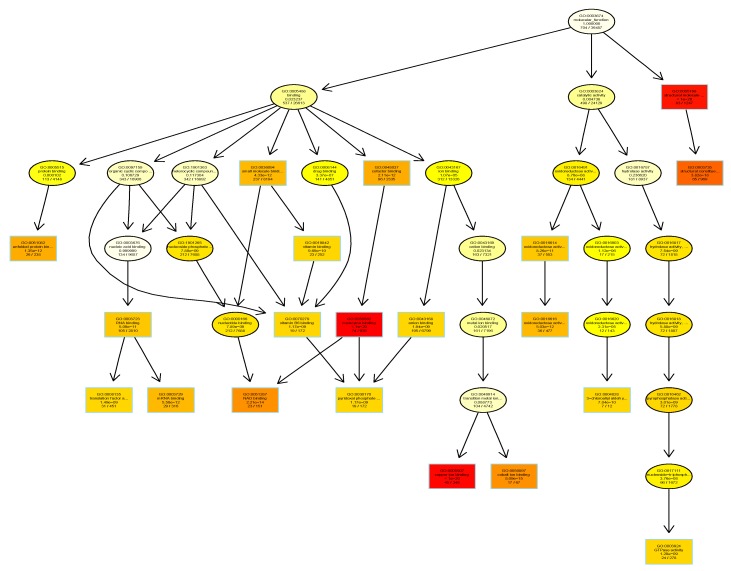
Gene ontology enrichment analysis using TopGO package in R. Gene Ontologies (GOs) associated with molecular function category in barley proteins identified in the kilned rootlets is shown here. Each box shows the GO term number, brief description and the p-value. The first pair of numerals represents the number of proteins in the input list associated with that GO term and the second numeral represents the total number of proteins in the barley proteome associated with that term. The box color indicates statistical significance with yellow = 1 × 10^−3^, orange = 1 × 10^−5^, and red = 1 × 10^−7^.

GO annotations for the entire barley genome were determined based on the GOMAP [18] (https://github.com/Dill-PICL/GOMAP-singularity) strategy for high-coverage, high-confidence annotation set, while using sequence similarity and protein domain presence methods as well as mixed-method pipelines described previously [19]. Based on this integrated strategy, 39,734 genes were found to have GO annotations [18] (https://github.com/Dill-PICL/GOMAP-singularity). In comparison, the version 2 of the AgriGO database the number of barley genes with GO annotations is less than 22,000 genes [20]. Almost all the genes in the barley database were associated with one or more terms in the “Biological Process” (BP) category. Barley genes were assigned to 7838 BP terms, with 3341 terms associated with “Molecular Function” (MF) and 1331 terms associated with “Cellular Component” (CC). The topGO package in Bioconductor was used to identify the enriched gene ontologies associated with the rootlet proteome in RStudio. The barley GOMAP entries were used as the background. The list of unique 683 proteins from the barley rootlet proteome was assigned to 5321, 2849, and 1072 GO terms associated with BP, MF, and CC, respectively. Enrichment analysis identified that there are 177 BP terms (Figure S1), 68 MF terms (Figure 3), and 60 CC terms that are statistically significantly higher in the kilned rootlet proteome (Figure S2).

### 2.5. Pathway Enrichment Analysis of the Rootlet Proteome

There are 274 common proteins and 95 proteins unique to the kilned sample, thus giving a total of 369 proteins as being in the rootlets but not in the seeds, based on the germinating rootlets versus kilned rootlets comparison. A careful examination of these 369 gene identifiers indicated that it corresponded to 364 unique proteins. 

**Figure 4:**
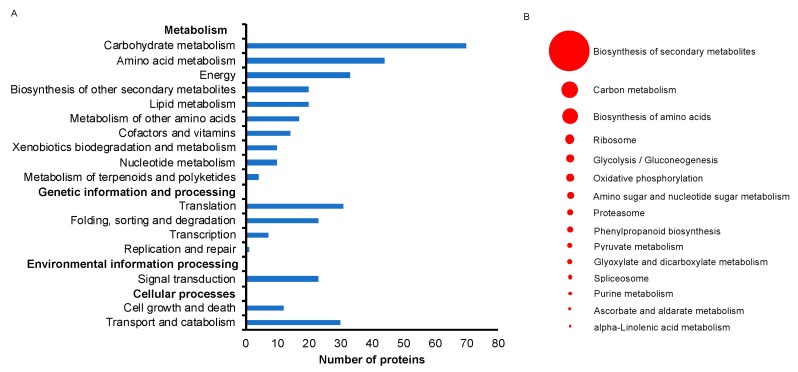
Kellogg Encyclopedia of Genes and Genomes (KEGG) pathway analysis of identified proteins from rootlets. (**A**) Major KEGG pathways for the identified proteins are presented. (**B**) The most abundant 15 KEGG sub pathways pathways are highlighted. The size of the circular ring, ranging from 5 to 67, stands for the number of proteins involved in these pathways.

The 364 unique protein identifiers accounting for 3166 transcripts were subjected to BLASTKOALA [21] to obtain their corresponding K-numbers to further investigate the biological function of the proteins in the rootlets. Of the 3166 entries, 2203 (69.6%) were annotated. This provided a list of 348 unique K numbers that was then used for Kellogg Encyclopedia of Genes and Genomes (KEGG) mapping [22]. These 348 K numbers were mapped to 184 KEGG pathways (Table S5) and 30 KEGG Brite terms (Table S6). Of the 17 main pathways (Figure 4A), 10 were associated with metabolism, four were associated with genetic information and processing, two with cellular processes, and one with environmental information processing. Of the 15 sub pathways that consisted of more than five proteins, the biosynthesis of secondary metabolites was predominant (Figure 4B). Ascorbate metabolism and alpha-linolenic acid metabolism were two other unique categories.

**Figure 5:**
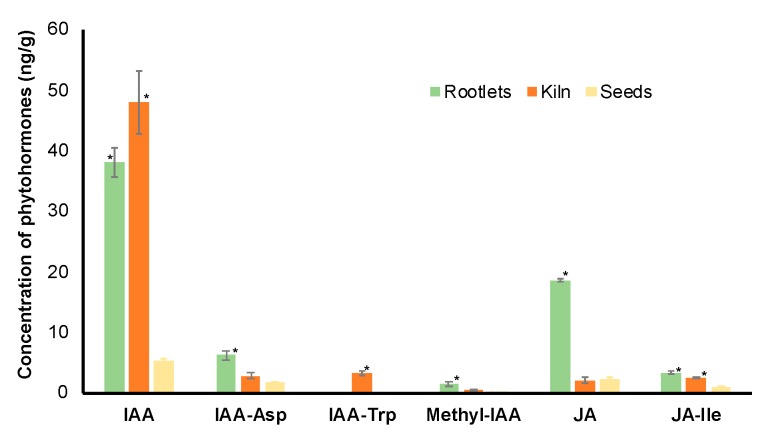
Analysis of free and conjugated forms of auxin and jasmonic acid (JA) in the dry seeds, kilned seeds and rootlets of barley. * represents statistical significance of the values (*p* < 0.05) in comparison with values in dry seeds.

There is a plethora of literature demonstrating a vital role for the phytohormones in the root developmental processes [23]. KEGG pathway analysis identified the linolenic acid metabolic pathway that was associated with JA biosynthesis was enriched (Figure S3). Interestingly, the Plant Reactome database analysis identified the auxin biosynthesis and transport pathways among the proteins in the rootlets (Figure S4). Based on these observations, the levels of these two phytohormones and their conjugated forms were examined in fresh seeds, kilned seeds, and rootlets (Figure 5).

For both IAA and JA, the free form of the hormones was most abundant in the rootlets. It is interesting to note that the levels of IAA are significantly higher in the kilned seeds when compared to the fresh seeds.

**Figure 6:**
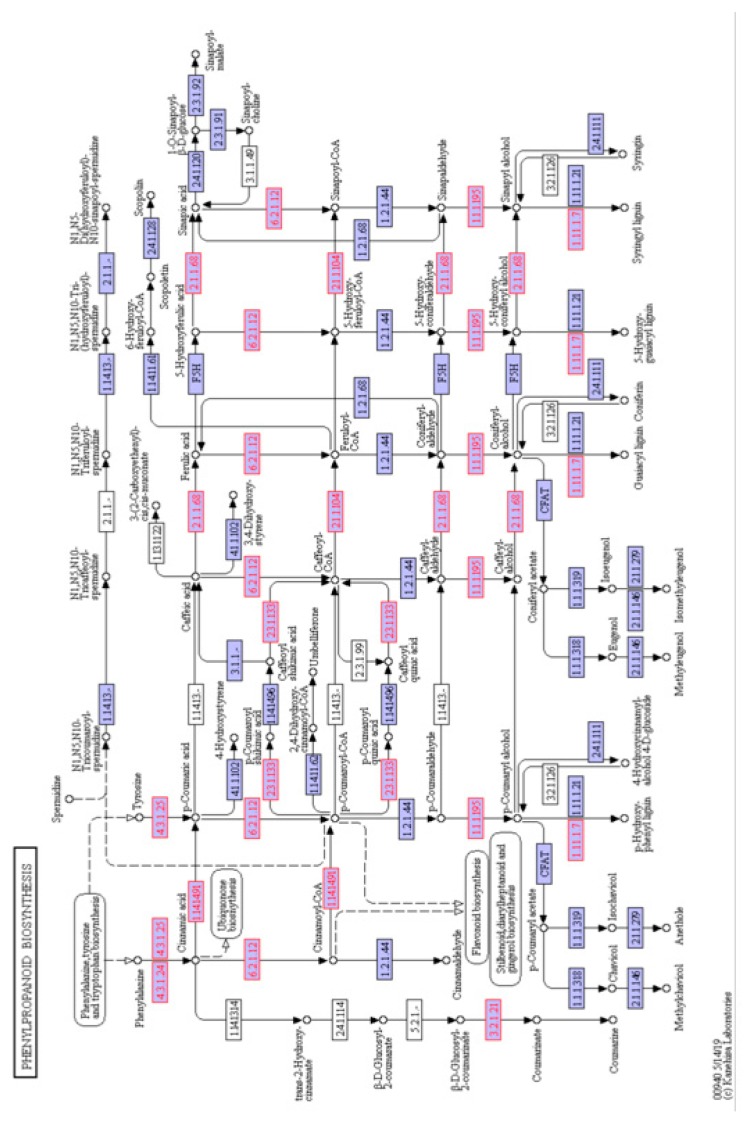
Schematic of the phenylpropanoid biosynthesis pathway from the KEGG database. The enzyme numbers shown in red font represent the proteins that were identified in the barley rootlets. Purple box represent other enzymes in the pathway identified in other plants.

There were 33 enzymes associated with phenylpropanoid metabolism that were identified in the rootlets (Figure 6). Branch pathways leading to production of coumarine, sinapoyl-CoA, Feruloyl-CoA, and various types of lignin molecules were identified.

## 3. Discussion

Temporal analyses of the barley proteome during five different stages of malting were reported earlier [2]. However, the samples that were used in this earlier study comprised of both the seeds and the rootlets. In the industrial malting process, the rootlets are usually separated from the kilned seeds and the seeds (without the rootlets) are used in the subsequent mashing and fermentation steps to produce beer. The rootlets are byproducts of the malting industry and they are mostly used as animal feed. Gaining insight into the rootlet proteome was the primary goal of this study.

The total number of proteins identified in the rootlets was nearly 25% more than the number of proteins identified in the kilned seeds. The proteome complexity of the rootlets can be appreciated by taking a closer look at its anatomy. Based on the terminology outlined [24] for describing the various parts of the complex root system of barley, each cylinder of root tissue that develops from the seed or the stem is an axis. There are two distinct types of axis recognized in barley: seminal axes, which develop from initials in the embryo [25]; and, nodal axes, which develop later from the growing shoot and its tillers. The root tissues examined in this analysis mainly constitutes of seminal axes based on this anatomical description. As the first organ that emerges after germination, seminal axes provide water and nutrients for the growing seedling. The complexity of this seemingly simple tissue is revealed when considering the longitudinal structure of barley roots. It is characterized by a root cap at the terminal end, a subterminal meristematic zone, followed by zones in which newly formed cells elongate and differentiate [26]. Hence, roots represent a gradient of cell differentiation along the longitudinal axis: young and undifferentiated cells are located at the distal end near the root tip, whereas the differentiated cells are located toward the proximal end of the root. It is evident that in a rootlet tissue sample with cells of different stages of differentiation is more complex when compared to a seed tissue and this is reflected in the number of proteins that were identified from these tissues.

Radially, barley roots can be divided into the stele and the surrounding cortical parenchyma and piliferous layer. The endodermis bounding the stele comprises of tangentially elongated cells. Immediately within the endodermis lies the pericycle consisting of the vascular tissues—xylem, phloem, and the ground tissue. Given this anatomical complexity that entails multiple cell types, it is not surprising that a larger number of proteins were identified in the seminal axes when compared to the seeds.

Three main observations from the GO enrichment analysis highlight the key features of the rootlet proteome. Firstly, primary metabolism that includes Glycolysis/gluconeogenesis, TCA cycle, and pentose phosphate shunt, was enriched, suggesting that the production of sugars is one of the key processes in the rootlets. In fact, of the 73 known metabolites reported in germinating barley seeds, more than a dozen were sugars [27]. Furthermore, the enrichment of genes associated with these pathways has also been reported in transcriptome analysis of germinating rice and barley seeds [28] and storage roots of cassava [29]. It has been reported that, in developing seeds as well as in the roots the predominant flux is from sugars to phosphoenol pyruvate (PEP) and not via gluconeogenesis [30,31]. This begs the question, why is the gluconeogenesis pathway enriched during barley rootlet development? It is known that sugars predominantly provide the bulk of the PEP. At certain developmental stages and/or under certain ambient conditions (hypoxia), the carbon skeletons of amino acids/amides and/or organic acids provide PEP (such as malate and citrate). Under these latter conditions, gluconeogenesis would occur if the amount of PEP that was produced was in excess [32]. Where does this excess PEP come from? It is speculated that to produce this PEP there is either an enhanced metabolism of amino acids/ amides/organic acids and/or changes in flux through certain branches of the pathways by which they are metabolized. Consistent with this hypothesis, it is interesting to note that the cellular amine metabolic process and malate metabolic processes were also significantly enriched in the rootlet proteome. Gluconeogenesis has been implicated in the regulation of the concentration of metabolites and pH, avoidance of excessive O_2_ consumption, or CO_2_ production that may be necessary when large amounts of nitrogenous compounds are metabolized [33,34,35]. Nitrogenous compound metabolic processes were enriched in the rootlet proteome, lending further credence to these observations.

Secondly, the molecular function and cellular compartment GO terms analysis both identified the process of translation as a significant feature in the rootlet proteome. It was also observed that there was significant enrichment for the proteasome and the peptidase activity in the group of proteins identified in the rootlet proteome. The transcriptional upregulation of translation and proteasome pathway genes during barley germination has been reported [28]. Thus, new protein synthesis and protein degradation processes both occur simultaneously in the developing seminal roots of barley. One of the major reasons for consuming cereal sprouts has been attributed to the enhancement of its nutritive value by the process of germination that increases the protein concentration, amino acids, sugars, and vitamins [36]. Analysis of the proteome of the developing rootlets and the significant enrichment of GO associated with protein synthesis and protein breakdown lends further support to these studies.

Thirdly, it was observed that the terms that were associated with stress pathways were significantly enriched in the rootlet proteome. During the process of malting, the seeds are subjected to steeping regime, wherein they experience intermittent periods of hypoxia. This is followed by germination in canisters that is tightly packed (crowding stress) and it is subjected to continuous rotation (to slow down the growth), which further contributes to a stressful ambience for the developing rootlets. Given these conditions from which these rootlet samples were collected, it is not surprising to see proteins that are associated with stress responses are significantly enriched in their proteome.

The enrichment of ascorbate and aldarate metabolic pathway in the kilned rootlets suggests that it can be useful source of ascorbic acid commonly referred to as vitamin C. It is a dietary supplement with antioxidant properties [37], which is used for treating scurvy diseases, and is also important for boosting immune system function. Interestingly, analysis of the proteome data from kilned rootlets while using Plant Reactome database indicated the enrichment of the glutathione pathway. It is well known that glutathione is a useful antioxidant, and it is also involved in detoxifying chemicals that are naturally produced in the body, as well as pollutant and drugs [38]. The free extract from the malt rootlets were shown to have antioxidant activity [7] and the enrichment of the ascorbate and GSH pathways lends further support to this study.

Transcriptome analysis during barley seed germination suggested the upregulation of carbohydrate metabolism and protein degradation pathways coordinated by phytohormones, especially GA and ABA [39]. Interestingly, in the rootlet proteome analysis, the pathway for JA and IAA were enriched. Auxins have been shown to impact the seed sensitivity to ABA and, in turn, germination [40]. In the context of the rootlets, IAA has been shown to accumulate in the roots and it plays a central role in cell division and growth [41]. It is tempting to speculate that JA might be induced more in response to the stress during the malting process. Most importantly, the confirmation of higher quantities of these two phytohormone in the rootlets (Figure 5) renders credibility to the proteome data set and the pathway enrichment analysis. It also emphasizes the importance of conducting omics studies using specific tissue and/or cell types.

A careful examination of the phenylpropanoid pathway enzymes suggested that kilned rootlets could be good source of useful compounds, including coumarin, cinnamaldehyde, sinapic acid, and sinapyl alcohol (Figure 6). Coumarin has been associated with various biological activities, including anti-inflammatory, antifungal, antibacterial, and anti-tumor properties [42]. Many of the anticoagulants, such as warfarin, are a coumarin derivative [43]. Sinapic acid is capable of absorbing laser and donating a proton to the analyte of interest and it has hence found application as the matrix in the MALDI Mass spectrometry [44]. Sinapic acid has both antioxidant and antibacterial effects and, hence, a chemical compound for consideration as a preservative in foods, cosmetics, and the pharmaceutical industry [45]. Cinnamaldehyde has been mostly used as a flavoring agent in various food products [46] and it has also found application as a safe and effective insecticide against mosquito larvae [47].

Exploring recently developed MS1 ion current-based proteome analysis will lead to a more comprehensive repertoire of the proteins, although this study has reported on more than 800 proteins in the rootlet proteome [48]. One of the most important limitations in analyzing omics dataset is the availability of well-annotated genomes. In the KEGG pathway analysis, only about 70% of the barley entries were annotated, while in the Plant Reactome database analysis less than 50% of the barley entries were mapped back to the rice genome. Updating the barley database with well curated annotations linking to gene identifiers of model systems, like rice and Arabidopsis, will enable a deeper understanding of these types of datasets and facilitate more precise and insightful pathway mappings.

## 4. Materials and Methods

### 4.1. Plant Materials and Malting

Seeds of the malting barley variety Conrad were used for this study. The details of the malting procedures were described earlier [2]. The samples collected from one, three, five days after germination were placed in a strainer under liquid nitrogen and gently crushed using a metal spoon, so that the rootlets were separated from the hard seeds. Rootlets from one, three, and five days after germination were pooled together for proteome analysis. Following the kilning procedure [2], the rootlets from the kilned samples were removed, as described above. 

### 4.2. Protein Extraction

Approximately one gram of barley samples (roots or seeds) were placed in a pre-cooled mortar and then ground to a fine powder with pestle. Protein isolations, enzymatic “in Liquid” digestion and NanoLC-MS/MS were conducted, as described earlier [49]. The protein pellet was solubilized in a urea buffer pH 8.5 (8 mol L^−1^ urea in 50 mmol L^−1^ NH_4_HCO_3_) while using 100 μL of buffer/mg weight of pellet.

### 4.3. Enzymatic “In Liquid” Digestion

Extracted protein (200 μg) was TCA/acetone precipitated (9% TCA, 28% acetone final concentration) and the pellet re-solubilized and denatured in 30 μL of 8 M urea/50 mM NH_4_HCO_3_ (pH 8.5)/1 mM Tris–HCl for 5 min. Subsequent reduction, alkylation, and tryptic digestion were conducted, as described earlier [49]. Fifty micrograms of digested proteins (1/4th digestion volume) was cleaned up using OMIX C18 SPE cartridges (Agilent, Palo Alto, CA, USA) as per manufacturer protocol and eluted in 20 μL of 60/ 40/0.1% ACN/H_2_O/TFA, dried to completion in the speed-vac, and finally reconstituted in 50 μL of 0.1% formic acid.

### 4.4. Mass Spectrometry

The peptides were analyzed by nanoLC-MS/MS using the Agilent 1100 nanoflow system (Agilent, Palo Alto, CA, USA) connected to a new generation hybrid linear ion trap-orbitrap mass spectrometer (LTQ-Orbitrap Elite™, Thermo Fisher Scientific, Waltham, MA, USA) equipped with an EASY-Spray™ electrospray source at the University of Wisconsin Mass Spectrometry and Proteomics facility. The chromatography of peptides prior to mass spectral analysis was accomplished using a capillary emitter column (PepMap^®®^ C18, 3 μM, 100 Å, 150 Å ~ 0.075 mm, Thermo Fisher Scientific, Waltham, MA, USA) onto which 1 μL of extracted peptides was automatically loaded. The nanoHPLC system delivered solvents A: 0.1% (*v*/*v*) formic acid, and B: 99.9% (*v*/*v*) acetonitrile, 0.1% (*v*/*v*) formic acid. The peptides were loaded at 0.50 μL/min. over a 30-min. period and then eluted at 0.3 μL/min directly into the nano-electrospray with gradual gradient from 3% (*v*/*v*) B to 20% (*v*/*v*) B over 154 min The elution process concluded with 12-min fast gradient from 20% (*v*/*v*) B to 50% (*v*/*v*) B, at which time a 5-min flash-out from 50–95% (*v*/*v*) B took place. As peptides eluted from the HPLC-column/ electrospray source, survey MS scans were acquired in the Orbitrap with a resolution of 120,000, followed by MS2 fragmentation of 20 most intense peptides detected in the MS1 scan from 300 to 2000 m/z. Dynamic exclusion limited redundancy.

### 4.5. MS Data Analysis

Raw MS/MS data were converted to Mascot generic format (mgf) files while using MSConvert (ProteoWizard: Open Source Software for Rapid Proteomics Tools Development). The resulting mgf files were used to search against Uniprot’s Barley (*Hordeum vulgare*) database with decoy reverse entries (124,660 total entries) using in-house Mascot search engine 2.2.07 (Matrix Science, London, UK) with fixed carbamidomethylation on cysteine, plus variable methionine oxidation and asparagine/glutamine deamidation. Peptide mass tolerance was set at 15 ppm and fragment mass at 0.6 Da. Protein annotations, significance of identification, and spectral based quantification were done with the help of Scaffold software version 4.4.1 (Proteome Software Inc., Portland, OR, USA). The protein identifications were accepted if they could be established at greater than 99.0% probability within 1% False Discovery Rate (FDR) and contained at least two identified peptides. The Protein Prophet algorithm assigned protein probabilities [50]. Proteins that contained similar peptides and could not be differentiated based on MS/MS analysis alone were grouped to satisfy the principles of parsimony.

### 4.6. GO Enrichment Analysis Using TopGO

Sequence similarity and protein domain presence methods as well as mixed-method pipelines that included Argot2 (http://www.medcomp.medicina.unipd.it/Argot2), FANN-GO [51], and PANNZER (http://ekhidna.biocenter.helsinki.fi/pannzer) that was used for maize was implemented in order to obtain high coverage and high confidence GO annotations for the barley genome [19] (10.25739/zvgv-8e37). The TopGO package (https://bioconductor.org/packages/topGO/) from Bioconductor was implemented in RStudio to identify enriched GO associated with rootlets. The barley GO annotations retrieved from GOMAP (https://github.com/Dill-PICL/GOMAP-singularity) were used in the enrichment analysis using TopGO. Default settings were used for declaring significantly enriched terms.

### 4.7. Pathway Analysis Using KEGG and Plant Reactome

Sequences of 364 unique proteins in the kilned rootlets were subjected to BlastKOALA (https://www.kegg.jp/blastkoala/) to retrieve the K numbers for KEGG pathway analysis [21]. The KEGG genes database files of family_eukaryotes and genus_prokaryotes were used for the BLAST search. Pathway reconstruction of the retrieved mappings was executed in the KEGG database.

The rice orthologs of the barley proteins in the rootlets were retrieved from Ensembl (https://plants.ensembl.org/Hordeum_vulgare/Info/Index) while using the Biomart tool (https://plants.ensembl.org/biomart/martview). These rice gene identifiers were then used for pathway enrichment in Plant Reactome database [52].

### 4.8. Phytohormone Analysis

About 200 mg of the finely ground sample was weighed out, after vortexing and pipetting in and out several times to make sure the solution was homogenous, was dried down in a 2 mL Eppendorf tube and then extracted for hormones. Hormones were extracted while using cold methanol: acetonitrile (50:50, *v*/*v*) spiked with deuterium-labeled internal standards (mixture of D5-IAA, D2-JA at 1.25 μM each). The sample was shaken for 25 min and then centrifuged at 16,000× *g* for 10 min. The supernatant was transferred to a new tube and the extraction of the pellet was repeated one more time. The supernatants were pooled and dried down while using a speed-vac. The pellets were re-dissolved in 200 μL of 15% methanol.

For LC separation, ZORBAX Eclipse Plus C18 column (2.1 mm × 100 mm) (Agilent, Palo Alto, CA, USA) was used flowing at 0.45 mL/min. The gradient of the mobile phases A (0.1% formic acid) and B (0.1%formic acid /90% acetonitrile) was as follow: 5% B for 1 min, to 60% B in 4 min, to 100% B in 2 min, hold at 100% B for 3 min, to 5% B in 0.5 min. The Shimadzu LC system (Shimadzu, Kyoto, Japan) was interfaced with a Sciex QTRAP 6500+ (SCIEX, Farmingham, MA, USA) mass spectrometer that was equipped with a TurboIonSpray (TIS) electrospray ion source. Analyst software (version 1.6.3) (AB SciEx Pvt. Ltd., Singapore) was used to control the sample acquisition and data analysis. The QTRAP 6500+ mass spectrometer was tuned and calibrated according to the manufacturer’s recommendations. The hormones were detected while using MRM transitions that were optimized using standards. The instrument was set-up to acquire in positive and negative ion switching. For quantification, an external standard curve was prepared using a series of standard samples that contained different concentrations of unlabeled hormones and fixed concentrations of the deuterium-labeled standards mixture. The data were normalized based on the internal standards: D5IAA, D2JA, to account for experimental variation and hormone extraction/ionization efficiency. The amounts in ng/g of the hormones detected are reported.

### 4.9. Statistical Analysis

The student *t*-test was used determine if the mean of the phytohormone quantities were significantly different (*p < 0.05*) between the three samples (dry seeds, kilned rootlets, and seeds).

## Figures and Tables

**Table 1:** Summary of the replicate Liquid chromatography mass spectrometry/MS (LC-MS/MS) runs using barley rootlets and kilned seeds.

Sample	No. of Spectra		No. Peptides		Peptides		Proteins	
	R1	R2	R1	R2	Average	FDR (%)	Average	FDR (%)
Kiln seeds	43,698	43,839	10,851	12,048	11,450	0.26	1330	0.54
Rootlets	45,419	42,262	15,768	13,320	14,544	0.33	1668	0.60
Kiln rootlets	45,758	45,651	15,740	15,142	15,441	0.33	1694	0.52

R1 and R2 denote biological replicates with one or more spectral counts in both biological replicates.

## Supplementary Materials

Supplementary materials can be found at https://www.mdpi.com/1422-0067/21/1/179/s1. Table S1. List of all the proteins identified in barley rootlets and kilned seeds; Table S2. List of 765 proteins identified in barley germinating rootlets; Table S3. List of 805 proteins identified in kilned rootlets of barley; Table S4. List of 587 proteins identified in kilned barley seeds; Table S5. List of KEGG pathways associated with the barley rootlets; Table S6. List of KEGG BRITE terms associated with barley rootlets; Figure S1. GO enrichment analysis for the “Biological Process” category associated with barley rootlets; Figure S2. GO enrichment analysis for the “Cellular Compartment” category associated with barley rootlets; Figure S3. KEGG pathway for linoleic acid metabolism associated with barley rootlets; Figure S4. A. Auxin biosynthesis and B. Auxin transport pathways associated with barley rootlets identified from the Plant Reactome database.

## Abbreviations

GO	Gene Ontology
BP	Biological Process
MF	Molecular Function
PEP	Phosphoenol pyruvate
IAA	Indole acetic acid
JA	Jasmonic acid
MS	Mass Spectrometry
KEGG	Kellogg Encyclopedia of Genes and Genomes
LC-MS	Liquid chromatography mass spectrometry
mgf	Mascot Generic Format
FANN	Functional annotator
PANNZER	Protein Annotation with Z-score
Argot2	Annotation retrieval of gene ontology terms
