# Supplementary material for: Analysis of the Barley Malt Rootlet Proteome
Source: Int J Mol Sci. 2019 Dec 26;21(1):179. doi: 10.3390/ijms21010179 (PMC6981388; doi:10.3390/ijms21010179)
Supplement: Supplementary file 1 [file ijms-21-00179-s001.zip › Table S5_121619 .pdf]

Table S5. List of KEGG pathways associated with the unique proteins found in the kilned rootlets.

Metabolism

Global and overview maps

- 01100 Metabolic pathways (115)
- 01110 Biosynthesis of secondary metabolites (67)
- 01120 Microbial metabolism in diverse environments (32)
- 01130 Biosynthesis of antibiotics (36)
- 01200 Carbon metabolism (27)
- 01210 2-Oxocarboxylic acid metabolism (4)
- 01212 Fatty acid metabolism (4)
- 01230 Biosynthesis of amino acids (25)
- 01220 Degradation of aromatic compounds (1)

Carbohydrate metabolism

- 00010 Glycolysis / Gluconeogenesis (13)
- 00020 Citrate cycle (TCA cycle) (4)
- 00030 Pentose phosphate pathway (4)
- 00040 Pentose and glucuronate interconversions (1)
- 00051 Fructose and mannose metabolism (4)
- 00052 Galactose metabolism (3)
- 00053 Ascorbate and aldarate metabolism (5)
- 00500 Starch and sucrose metabolism (5)
- 00520 Amino sugar and nucleotide sugar metabolism (10)
- 00620 Pyruvate metabolism (8)
- 00630 Glyoxylate and dicarboxylate metabolism (8)
- 00640 Propanoate metabolism (3)
- 00650 Butanoate metabolism (2)
- 00562 Inositol phosphate metabolism (1)

Energy metabolism

- 00190 Oxidative phosphorylation (13)
- 00710 Carbon fixation in photosynthetic organisms (8)
- 00720 Carbon fixation pathways in prokaryotes (3)
- 00680 Methane metabolism (5)
- 00910 Nitrogen metabolism (2)
- 00920 Sulfur metabolism (2)

Lipid metabolism

- 00061 Fatty acid biosynthesis (3)
- 00071 Fatty acid degradation (2)
- 00073 Cutin, suberine and wax biosynthesis (1)
- 00100 Steroid biosynthesis (3)
- 00561 Glycerolipid metabolism (1)
- 00590 Arachidonic acid metabolism (2)
- 00591 Linoleic acid metabolism (1)
- 00592 alpha-Linolenic acid metabolism (5)
- 01040 Biosynthesis of unsaturated fatty acids (2)

Nucleotide metabolism

- 00230 Purine metabolism (6)
- 00240 Pyrimidine metabolism (4)

Amino acid metabolism

- 00250 Alanine, aspartate and glutamate metabolism (8)
- 00260 Glycine, serine and threonine metabolism (7)
- 00270 Cysteine and methionine metabolism (7)
- 00280 Valine, leucine and isoleucine degradation (1)
- 00220 Arginine biosynthesis (5)
- 00330 Arginine and proline metabolism (5)
- 00340 Histidine metabolism (1)
- 00350 Tyrosine metabolism (2)
- 00360 Phenylalanine metabolism (2)
- 00400 Phenylalanine, tyrosine and tryptophan biosynthesis (6)

Metabolism of other amino acids

- 00410 beta-Alanine metabolism (4)
- 00430 Taurine and hypotaurine metabolism (1)
- 00450 Selenocompound metabolism (2)
- 00460 Cyanoamino acid metabolism (4)
- 00471 D-Glutamine and D-glutamate metabolism (1)
- 00480 Glutathione metabolism (5)

Metabolism of cofactors and vitamins

- 00730 Thiamine metabolism (1)
- 00750 Vitamin B6 metabolism (2)
- 00770 Pantothenate and CoA biosynthesis (1)
- 00780 Biotin metabolism (1)
- 00670 One carbon pool by folate (4)
- 00860 Porphyrin and chlorophyll metabolism (1)
- 00130 Ubiquinone and other terpenoid-quinone biosynthesis (4)

Metabolism of terpenoids and polyketides

- 00902 Monoterpenoid biosynthesis (2)
- 00906 Carotenoid biosynthesis (1)
- 01051 Biosynthesis of ansamycins (1)

Biosynthesis of other secondary metabolites

- 00940 Phenylpropanoid biosynthesis (9)
- 00945 Stilbenoid, diarylheptanoid and gingerol biosynthesis (2)
- 00941 Flavonoid biosynthesis (3)
- 00950 Isoquinoline alkaloid biosynthesis (1)
- 00960 Tropane, piperidine and pyridine alkaloid biosynthesis (1)
- 00333 Prodigiosin biosynthesis (1)
- 00999 Biosynthesis of secondary metabolites - unclassified (3)

Xenobiotics biodegradation and metabolism

- 00364 Fluorobenzoate degradation (1)
- 00361 Chlorocyclohexane and chlorobenzene degradation (1)
- 00623 Toluene degradation (1)
- 00643 Styrene degradation (1)
- 00980 Metabolism of xenobiotics by cytochrome P450 (1)
- 00982 Drug metabolism - cytochrome P450 (1)
- 00983 Drug metabolism - other enzymes (4)

Genetic Information Processing
Transcription

- 03040 Spliceosome (7)

Translation

- 03010 Ribosome (15)
- 00970 Aminoacyl-tRNA biosynthesis (5)
- 03013 RNA transport (7)
- 03015 mRNA surveillance pathway (2)
- 03008 Ribosome biogenesis in eukaryotes (2)

Folding, sorting and degradation

- 03060 Protein export (2)
- 04141 Protein processing in endoplasmic reticulum (5)
- 04120 Ubiquitin mediated proteolysis (1)
- 03050 Proteasome (10)
- 03018 RNA degradation (5)

Replication and repair

- 03420 Nucleotide excision repair (1)

Environmental Information Processing
Signal transduction

- 02020 Two-component system (2)
- 04010 MAPK signaling pathway (1)
- 04013 MAPK signaling pathway - fly (1)
- 04016 MAPK signaling pathway - plant (2)
- 04066 HIF-1 signaling pathway (7)
- 04020 Calcium signaling pathway (1)
- 04072 Phospholipase D signaling pathway (1)
- 04024 cAMP signaling pathway (1)
- 04022 cGMP-PKG signaling pathway (1)
- 04152 AMPK signaling pathway (2)
- 04150 mTOR signaling pathway (3)
- 04075 Plant hormone signal transduction (1)

Cellular Processes
Transport and catabolism

- 04144 Endocytosis (5)
- 04145 Phagosome (8)
- 04142 Lysosome (5)

- 04146 Peroxisome (2)
- 04140 Autophagy - animal (3)
- 04138 Autophagy - yeast (3)
- 04137 Mitophagy - animal (3)
- 04139 Mitophagy - yeast (1)

Cell growth and death

- 04210 Apoptosis (3)
- 04214 Apoptosis - fly (2)
- 04216 Ferroptosis (2)
- 04217 Necroptosis (4)
- 04218 Cellular senescence (1)

Cellular community - eukaryotes

- 04530 Tight junction (1)
- 04540 Gap junction (2)

Cellular community - prokaryotes

- 02024 Quorum sensing (3)
- 02026 Biofilm formation - Escherichia coli (1)

Cell motility

- 04810 Regulation of actin cytoskeleton (2)
