# Supplementary material for: Analysis of the Barley Malt Rootlet Proteome
Source: Int J Mol Sci. 2019 Dec 26;21(1):179. doi: 10.3390/ijms21010179 (PMC6981388; doi:10.3390/ijms21010179)
Supplement: Supplementary file 1 [file ijms-21-00179-s001.zip › Table S6_121619.pdf]

Table S6. List of KEGG BRITE terms associated with the unique proteins found in the kilned rootlets.

**Genes and Proteins**

Orthologs and modules

- ko00001 KEGG Orthology (KO) (244)

Protein families: metabolism

- ko01000 Enzymes (152)
- ko01001 Protein kinases (1)
- ko01009 Protein phosphatases and associated proteins (4)
- ko01002 Peptidases and inhibitors (15)
- ko01003 Glycosyltransferases (2)
- ko01004 Lipid biosynthesis proteins (5)
- ko01007 Amino acid related enzymes (8)
- ko00199 Cytochrome P450 (2)

Protein families: genetic information processing

- ko03021 Transcription machinery (1)
- ko03019 Messenger RNA biogenesis (13)
- ko03041 Spliceosome (11)
- ko03011 Ribosome (15)
- ko03009 Ribosome biogenesis (5)
- ko03016 Transfer RNA biogenesis (6)
- ko03012 Translation factors (10)
- ko03110 Chaperones and folding catalysts (6)
- ko04131 Membrane trafficking (22)
- ko04121 Ubiquitin system (3)
- ko03051 Proteasome (13)
- ko03036 Chromosome and associated proteins (9)
- ko03400 DNA repair and recombination proteins (1)
- ko03029 Mitochondrial biogenesis (7)

Protein families: signaling and cellular processes

- ko02000 Transporters (7)
- ko02044 Secretion system (2)
- ko04812 Cytoskeleton proteins (5)
- ko04147 Exosome (38)
- ko02048 Prokaryotic defense system (1)
- ko04040 Ion channels (1)
- ko04031 GTP-binding proteins (6)
