# Supplementary figures and images for: Analysis of the Barley Malt Rootlet Proteome
Source: Int J Mol Sci. 2019 Dec 26;21(1):179. doi: 10.3390/ijms21010179 (PMC6981388; doi:10.3390/ijms21010179)

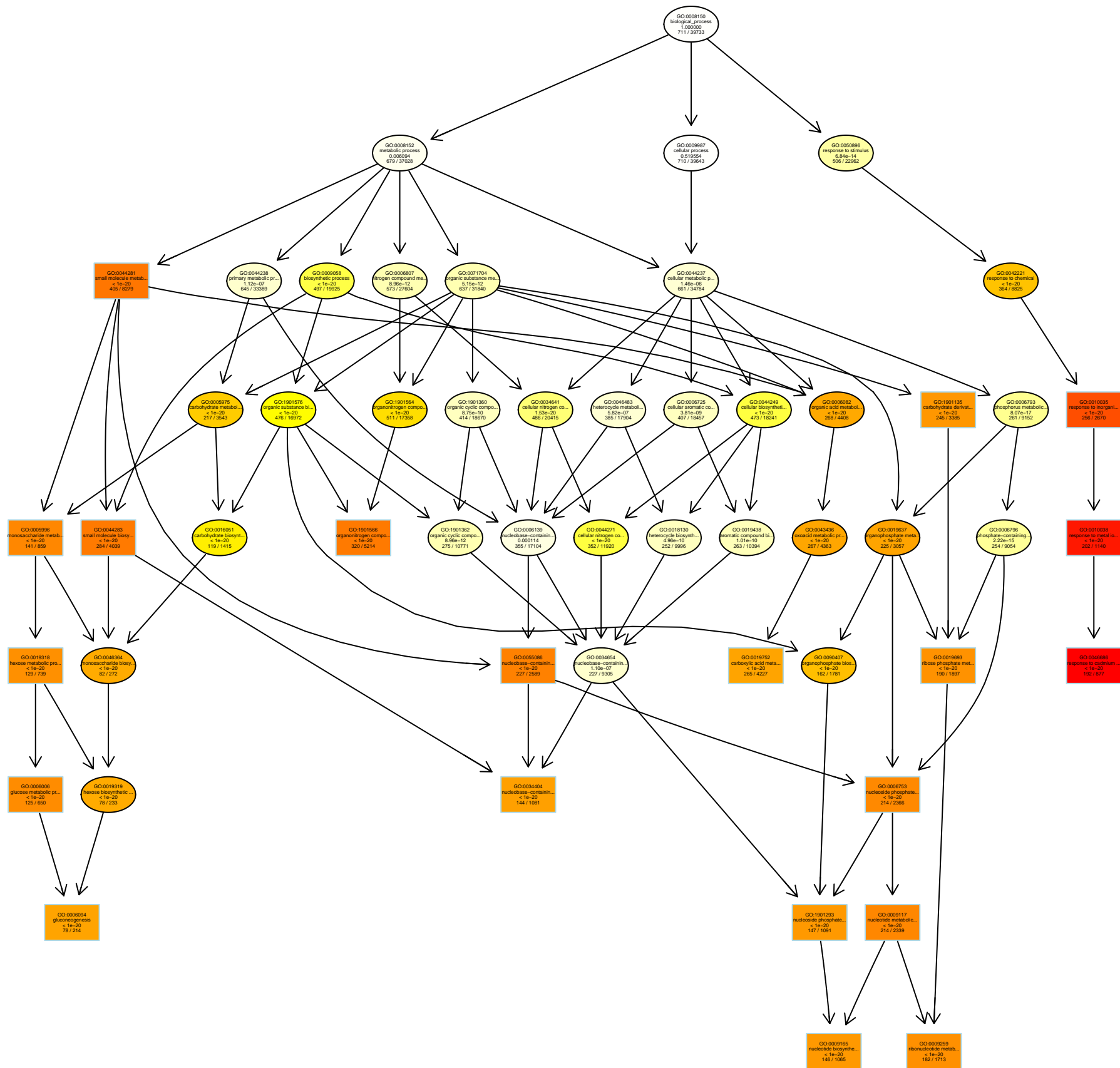

Supplement: Supplementary file 1 [file ijms-21-00179-s001.zip › Figure S1.pdf]

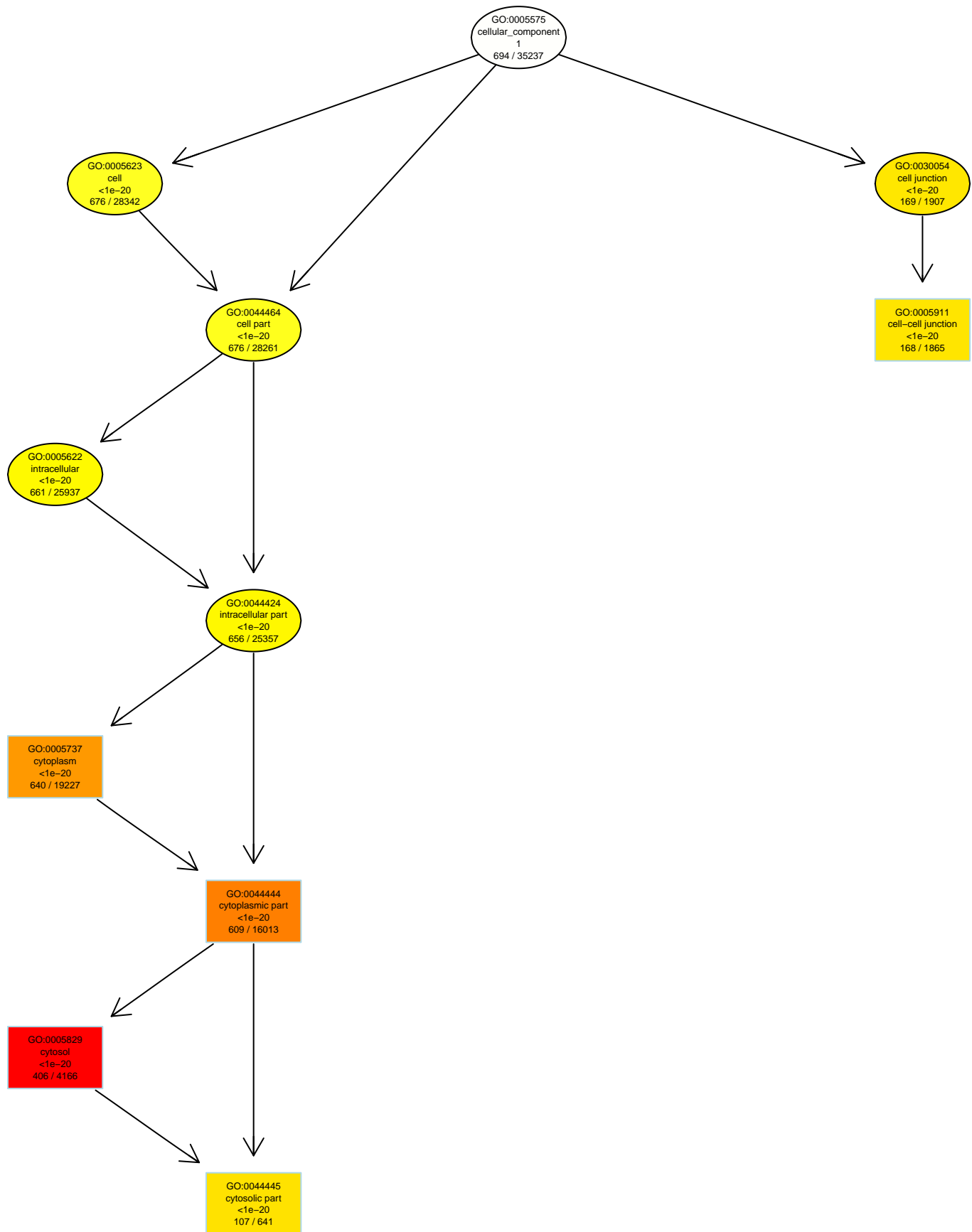

Supplement: Supplementary file 1 [file ijms-21-00179-s001.zip › Figure S2.pdf]

$\alpha$ -LINOLENIC ACID METABOLISM

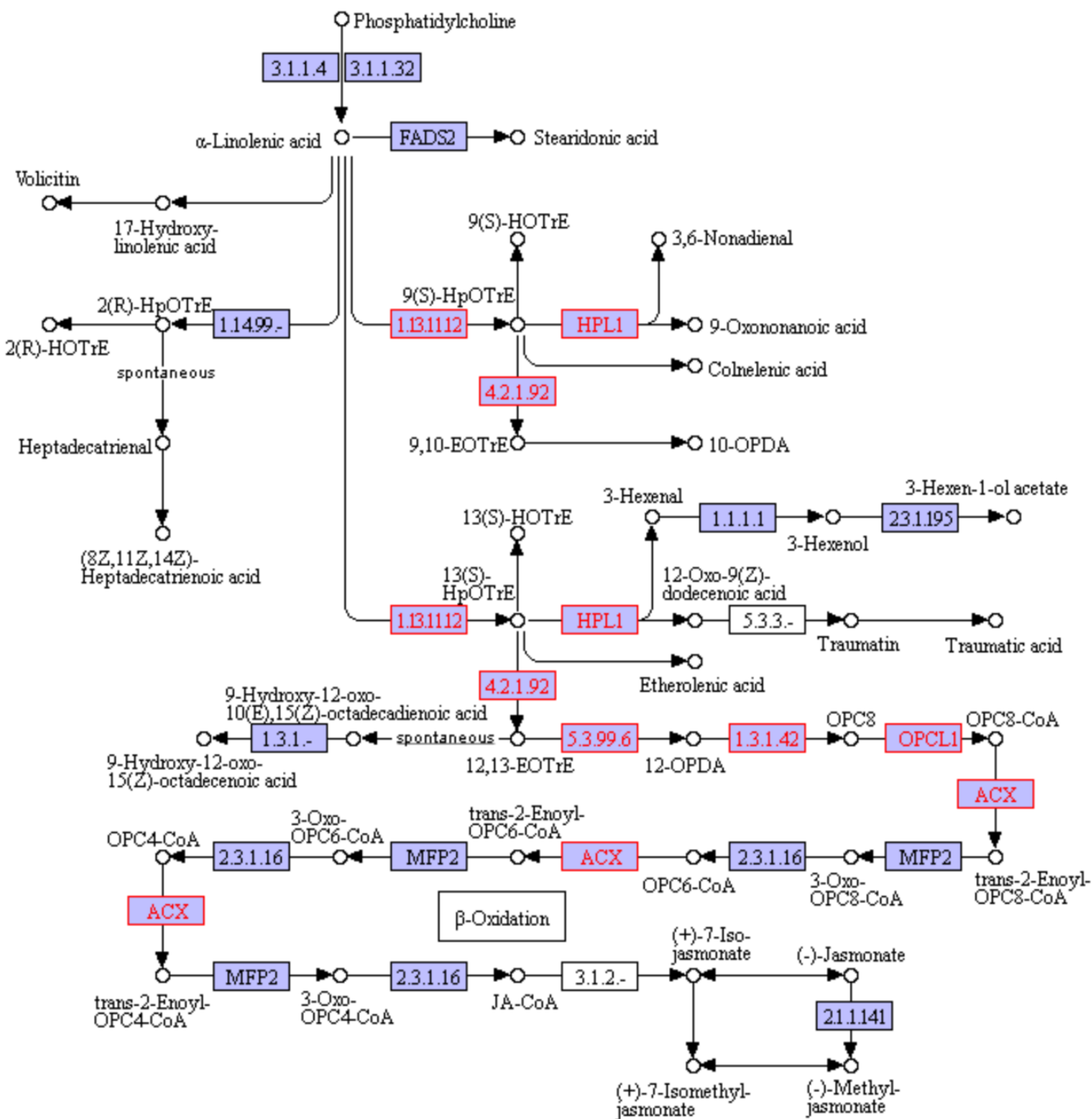

Supplement: Supplementary file 1 [file ijms-21-00179-s001.zip › Figure S3.pdf]

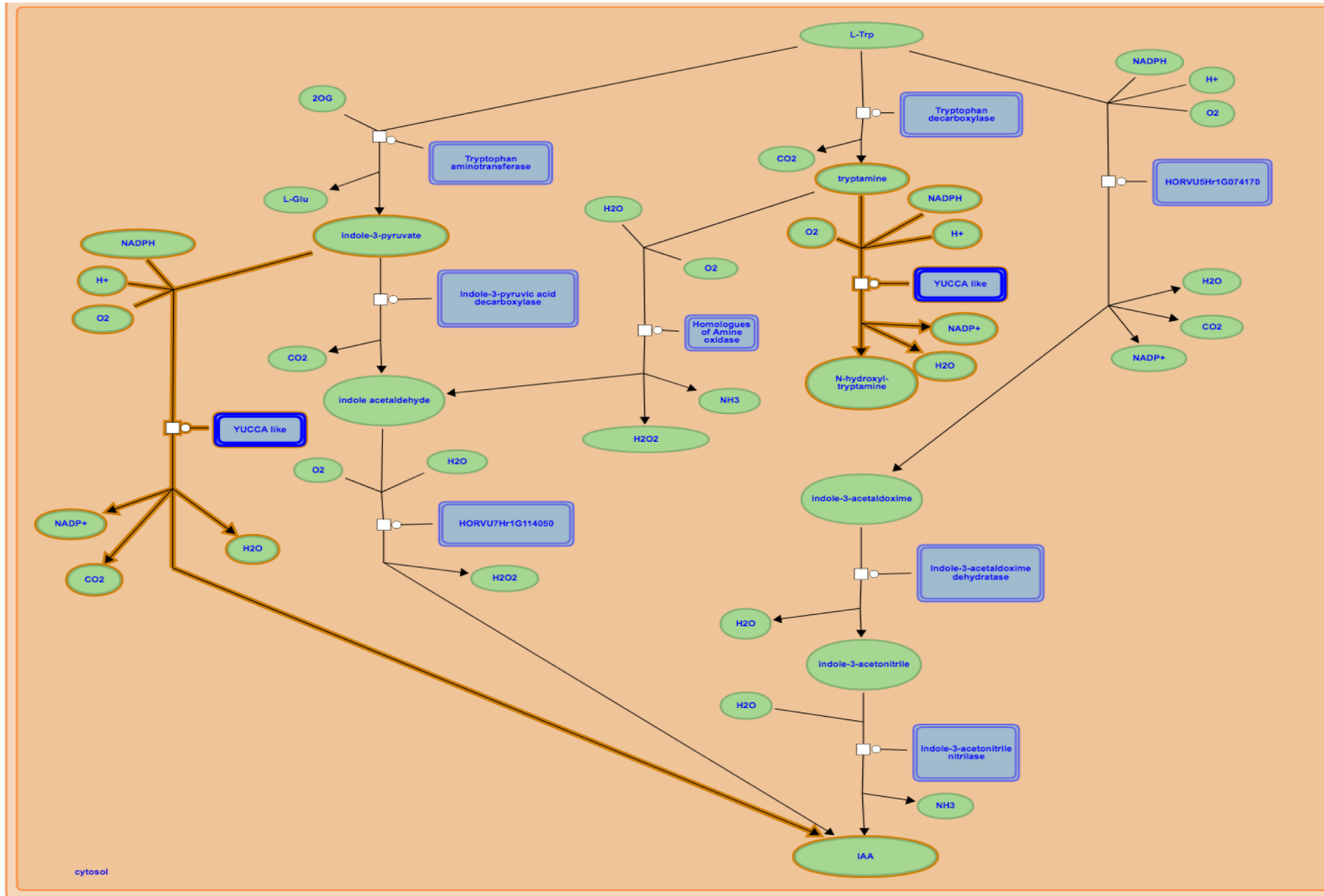

### Auxin biosynthesis pathway

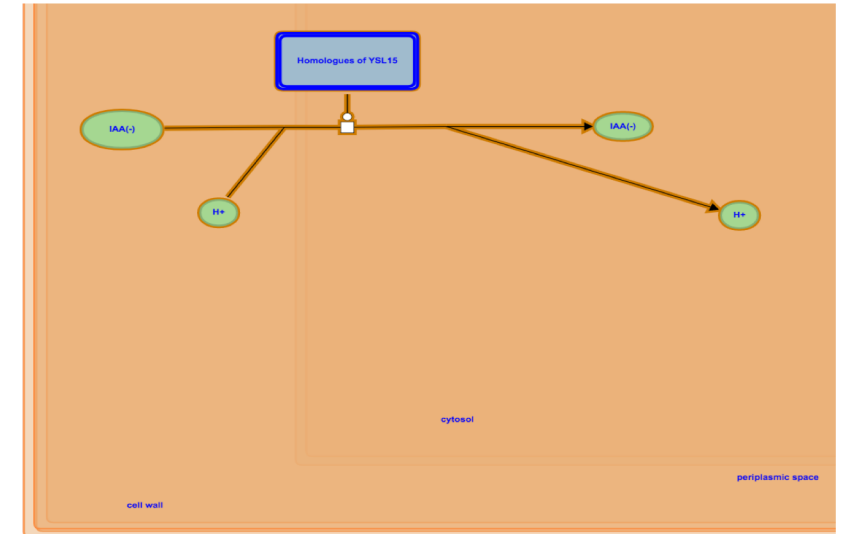

### Auxin transport pathway

Supplement: Supplementary file 1 [file ijms-21-00179-s001.zip › Figure S4.pdf]
